# Prognostic impact of CD8 and programmed death-ligand 1 expression in patients with resectable non-small cell lung cancer

**DOI:** 10.1038/s41416-019-0398-5

**Published:** 2019-02-12

**Authors:** Seok-Hyun Kim, Se-Il Go, Dae Hyun Song, Sung Woo Park, Hye Ree Kim, Inseok Jang, Jong Duk Kim, Jong Sil Lee, Gyeong-Won Lee

**Affiliations:** 1 Division of Hematology and Medical Oncology, Department of Internal Medicine, Samsung Changwon Hospital, Sungkyunkwan University School of Medicine, Changwon, 51353 Republic of Korea; 2 Division of Hematology-Oncology, Department of Internal Medicine, Gyeongsang National University Changwon Hospital, Gyeongsang National University School of Medicine, Changwon, 51472 Republic of Korea; 3 Department of Pathology, Gyeongsang National University Changwon Hospital, Gyeongsang National University School of Medicine, Changwon, 51472 Republic of Korea; 4 Division of Hematology-Oncology, Department of Internal Medicine, Gyeongsang National University Hospital, Gyeongsang National University School of Medicine, Jinju, 52727 Republic of Korea; 5 Department of Thoracic and Cardiovascular Surgery, Gyeongsang National University Hospital, Gyeongsang National University School of Medicine, Jinju, 52727 Republic of Korea; 6 Department of Pathology, Gyeongsang National University Hospital, Gyeongsang National University School of Medicine, Jinju, 52727 Republic of Korea

**Keywords:** Non-small-cell lung cancer, Immunoediting

## Abstract

**Background:**

The prognostic impact of the expression of CD8 and programmed death-ligand 1 (PD-L1) has not been established in patients with resectable non-small cell lung cancer (NSCLC).

**Methods:**

Surgical tissue specimens were obtained from 136 patients with NSCLC who underwent surgical resection. The expression levels of CD8 and PD-L1 were assessed using tissue microarrays and immunohistochemistry.

**Results:**

The CD8-positive group showed significant increases in overall survival (OS) (median, not reached [NR] vs. 28.452 months) and relapse-free survival (RFS) (median, NR vs. 14.916 months) compared with the CD8-negative group. In contrast to CD8, the PD-L1-negative group demonstrated significant increases in OS (median, NR vs. 29.405 months) and RFS (median, 63.573 vs. 17.577 months) compared with the PD-L1-positive group. Two prognostic groups were stratified according to CD8/PD-L1 expression: group 1 (CD8-positive/PD-L1-negative) vs. group 2 (CD8/PD-L1: positive/positive, negative/negative, negative/positive). Group 1 had better OS (median, NR vs. 29.405 months) and RFS (median, NR vs. 17.577 months) than group 2. Multivariate analysis indicated that group 1 constituted an independent favourable prognostic factor for OS (hazard ratio [HR], 0.329, *p* = 0.001) and RFS (HR, 0.293; *p* < 0.001).

**Conclusions:**

Positive CD8 and negative PD-L1 expression together may be favourable prognostic markers in resectable NSCLC.

## Introduction

Non-small cell lung cancer (NSCLC) accounts for approximately 80% of lung cancer cases.^1^ Among patients with NSCLC, approximately 43% have locoregional disease at the time of diagnosis.^2^ The 5-year survival rates of patients with resectable disease vary from 36 to 92% according to TNM stage.^3^ In addition to TNM stage, age, sex, type of surgery, and Glasgow prognostic score were reported to be prognostic factors in patients with resectable NSCLC.^4,5^ However, these factors are insufficient to predict patient survival, and more useful prognostic biomarkers are needed. Since immune checkpoint inhibitors have emerged as an optimal therapeutic option in advanced NSCLC,^6,7^ increased attention has been paid to immunologic biomarkers.

Currently, the predictive biomarkers of interest in this regard are the expression levels of programmed death-ligand 1 (PD-L1) and CD8.^8,9^ After activation, programmed death-1 (PD-1), a member of the CD28 costimulatory receptor superfamily, transmits inhibitory signals that abrogate T cell receptor (TCR)-mediated activating signals, preventing further antigen-mediated T-cell activation.^10^ The binding of PD-L1 to PD-1 induces apoptosis or exhaustion in activated T cells and limits the effector function of T cells in peripheral tissues during inflammatory responses.^11^ The PD-1/PD-L1 pathway is considered an important regulator of tumour-induced immune suppression, and blockade of the interaction has been found to promote the antitumour activity of T cells.^12^ PD-L1 is a potential biomarker to predict the treatment response and survival of advanced NSCLC patients treated with immune checkpoint inhibitors.^6,7^ However, there are debates about whether PD-L1 has a prognostic role in locoregional disease.^13–16^ Various cut-off values and methodologies to assess the expression of PD-L1 may partly explain this inconsistency. Furthermore, given that PD-L1 is inducible and may reflect homoeostatic responses to immune activation,^17^ immune cell infiltration should be considered simultaneously to assess the prognostic role of PD-L1 expression. Among the immunologic parameters, the expression of CD8, a marker for cytotoxic lymphocytes, has been most consistently reported as prognostic.^18–20^

Recently, several studies have assessed the clinical significance of PD-L1 and CD8 expression in localised and resectable NSCLC. However, inconsistent findings from positive to negative or insignificant results were reported for the prognostic value of these biomarkers.^13,21–24^ In this study, we suggest additional findings for clinical impact of expression of CD8 and PD-L1 as prognostic biomarkers in NSCLC patients treated with surgical resection.

## Methods

### Patients and tissue samples

Surgical tissue specimens from 136 patients with NSCLC who underwent surgical resection at Gyeongsang National University Hospital, Jinju, Korea, from October 2002 to January 2010 were obtained. This retrospective study was approved by the Institutional Review Board, which waived the requirement for informed consent. No patient received neoadjuvant chemotherapy or radiotherapy prior to thoracic surgery. Clinical stage was determined according to the seventh edition of the American Joint Committee on Cancer TNM staging system.^25^ Clinical characteristics were retrieved from available electronic medical records.

### Tissue microarray (TMA) and immunohistochemistry (IHC)

Representative tumour areas were marked on haematoxylin and eosin-stained slides and used for TMA construction. Tissue cores with a diameter of 3 mm were taken from donor paraffin blocks and placed in blank recipient paraffin blocks. Two cores per tumour were arrayed. The TMA blocks were cut into 4-μm sections, which were attached onto coated slides, labelled, and then placed on the Ventana Benchmark XT (Roche-Ventana, Tucson, AZ, USA). Sections were deparaffinised and subjected to pre-treatment with cell conditioning 1 solution (CC1, Roche-Ventana) for 60 min at 100 °C. Sections were then washed with reaction buffer followed by incubation with primary antibodies for 32–60 min at 37 °C. For PD-1 and CD8, rabbit anti-PD-1 monoclonal antibody (mAb) (clone E1L3 N, Cell Signalling Technology, Danvers, MA, USA; 1:200) and rabbit anti-CD8 mAb (clone SP16, Thermo Fisher Scientific, Fremont, CA, USA; 1:400) were used, respectively. Immunohistochemical staining was performed with the Ventana BenchMark XT autostainer (Ventana Medical Systems).

All IHC results were evaluated by two independent pathologists who were blinded to patients’ clinical outcomes. PD-L1 expression was calculated as the percentage of membrane staining on tumour cells with any intensity. Cases were considered positive when ≥1% of the tumour cells expressed PD-L1 (Supplement Figure S1). In the case of CD8, we calculated the positivity of CD8 using the Genie analysis tool (Leica Biosystems, Wetzlar, Germany). The absolute numbers of CD8-positive cells were automatically counted. The mean (±SD) and median (range) absolute number of CD8-positive cells were 752 (±697) and 543 (33–3460) per mm^2^, respectively. The cut-off value for CD8 expression was determined as the median absolute number and cases were considered positive when CD8-positive cells >543 per mm^2^ were observed.

### Statistical analysis

The association between the expression of each marker and clinicopathological parameters was analysed using the Mann–Whitney *U* or Kruskal–Wallis tests for CD8 data and *χ*^2^ test or Fisher’s exact tests for PD-L1 data. Survival probability analyses were performed using the Kaplan–Meier method. Long-term survivors were censored at 7 years of follow-up. Median follow-up duration was calculated by the reverse Kaplan–Meier approach. Overall survival (OS) was calculated from the date of surgery to the date of death from any cause or the date of the last follow-up observation. Relapse-free survival (RFS) was calculated from the date of surgery to the date of recurrence or the date of death from any cause. Kaplan–Meier analysis and log-rank tests were used to compare postoperative survival curves between groups. Univariate and multivariate analyses of survival were conducted using the Cox proportional hazards model with the enter selection method. Potential prognostic factors in the univariate analysis with *p* < 0.1 were included in the multivariate analysis. *p* values <0.05 were considered statistically significant. All analyses were performed using STATA 14.0 (Stata Corporation, College Station, Texas, USA) and R 3.4.0 (R Foundation for Statistical Computing, Vienna, Austria).

## Results

### Patients’ characteristics

This study included 136 patients with tissue-confirmed resected NSCLC. The median age at diagnosis was 66 (range 31–77) years and 116 (85.3%) patients were men. The most prevalent histology was squamous cell carcinoma (SqCC, 90/136, 66.2%). The majority of patients (124/136, 91.2%) had stage I/II disease, and adjuvant treatment was performed in 38 (27.9%) patients. A list of patients’ characteristics based upon CD8 and PD-L1 expression is shown in Table 1. In terms of histology, the proportion of SqCC was higher in the PD-L1-positive group than in the PD-L1-negative group (85.7% vs. 59.4%, *p* = 0.005). Otherwise, there were generally no significant differences in patients’ characteristics between groups except in the variables with unbalanced proportion such as gender and surgery type.

**Table 1:** Patients’ characteristics according to CD8 and PD-L1 expression

Variables	Group	*n*	CD8		PD-L1		
			Mean ± SD, cells/mm^2^	*p*	Negative *n* = 101	Positive *n* = 35	*p*
Gender	Male	116	758 ± 703	0.961	81 (80.2)	35 (100)	0.004
	Female	20	715 ± 676		20 (19.8)	0 (0)	
Age (years)	<65	59	723 ± 724	0.615	45 (44.6)	56 (55.4)	0.639
	≥65	77	774 ± 679		14 (40.0)	21 (60.0)	
Smoking	Never smoker	46	705 ± 632	0.786	34 (33.7)	12 (34.3)	0.947
	Smoker/ex-smoker	90	776 ± 730		67 (66.3)	23 (65.7)	
Histology	SqCC	90	755 ± 752	0.198	60 (59.4)	30 (85.7)	0.005
	Non-SqCC^a^	46	746 ± 580		41 (40.6)	5 (14.3)	
ECOG PS	0	103	735 ± 673	0.767	74 (73.3)	29 (82.9)	0.254
	1	33	803 ± 774		27 (26.7)	6 (17.1)	
Surgery	Lobectomy and others^b^	121	779 ± 674	0.011	89 (88.1)	32 (91.4)	0.759
	Pneumonectomy	15	533 ± 854		12 (11.9)	3 (8.6)	
TNM stage	I	76	729 ± 595	0.068	56 (55.5)	20 (57.1)	0.750
	II	48	826 ± 770		37 (36.6)	11 (31.4)	
	III	12	601 ± 980		8 (7.9)	4 (11.4)	
Adjuvant treatment	No treatment	98	779 ± 728	0.536	72 (71.3)	26 (74.3)	0.706
	Chemotherapy	16	717 ± 610		11 (10.9)	5 (14.3)	
	Concurrent chemoradiotherapy	15	571 ± 626		13 (12.9)	2 (5.7)	
	Radiotherapy	7	839 ± 634		5 (5.0)	2 (5.7)	
Relapse pattern	Local	29	620 ± 663	0.760	21 (61.8)	8 (57.1)	0.766
	Distant	19	779 ± 985		13 (38.2)	6 (42.9)	

Values are presented as number (%) for PD-L1

*SqCC* squamous cell carcinoma, *Non-SqCC* non-squamous cell carcinoma, *ECOG PS* Eastern Cooperative Oncology Group performance status

^a^Including adenocarcinoma, large cell carcinoma, bronchoalveolar carcinoma, and non-small cell carcinoma

^b^Bilobectomy (*n* = 1) and sleeve operation (*n* = 1)

### Survival analysis according to CD8 and PD-L1 expression

**Fig. 1:**
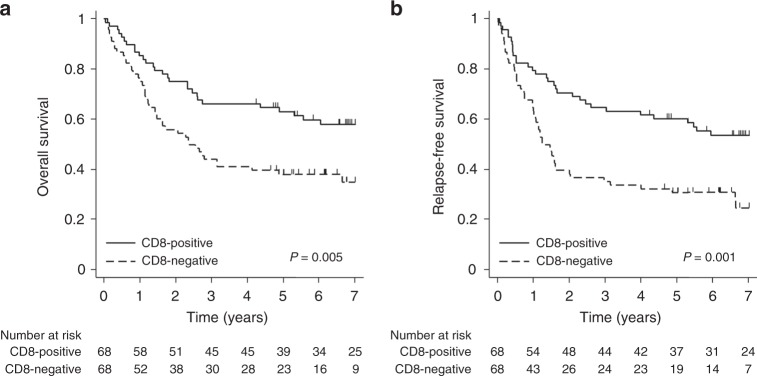
Kaplan–Meier curves for survival based on the expression of CD8 in resectable non-small-cell lung cancer. **a** Overall survival. **b** Relapse-free survival

**Fig. 2:**
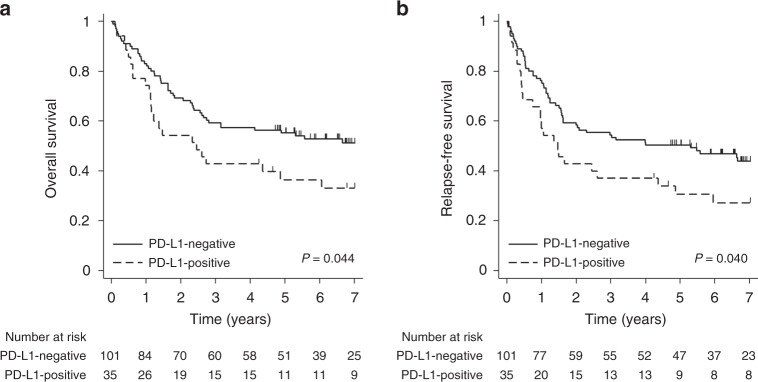
Kaplan–Meier curves for survival based on the expression of PD-L1 in resectable non-small-cell lung cancer. **a** Overall survival. **b** Relapse-free survival

In the entire patient group, the median OS and RFS were 63.573 months (95% confidence interval [CI], 31.212—not reached [NR]) and 35.647 months (95% CI 19.023–79.507), respectively, at a median follow-up of 84 months. The CD8-positive group (median OS, NR; 95% CI 63.573 months—NR) showed a significant increase in OS compared with the CD8-negative group (median OS, 28.452 months; 95% CI 17.019–58.349; *p* = 0.005, Fig. 1a). RFS was also significantly increased in the CD8-positive group (median RFS, NR; 95% CI 47.836 months—NR) compared with the CD8-negative group (median RFS, 14.916 months; 95% CI 12.058–23.984; *p* = 0.001, Fig. 1b). In contrast to CD8, the PD-L1-negative group (median OS, NR; 95% CI 33.511 months—NR) showed a significant increase in OS compared with the PD-L1-positive group (median OS, 29.405 months; 95% CI 13.667–72.509; *p* = 0.044, Fig. 2a). RFS was also significantly increased in the PD-L1-negative group (median RFS, 63.573 months; 95% CI, 19.450—NR) compared with the PD-L1-positive group (median RFS, 17.577 months; 95% CI, 8.871–52.238; *p* = 0.040, Fig. 2b).

### Survival analysis using the combined model of CD8 and PD-L1

**Fig. 3:**
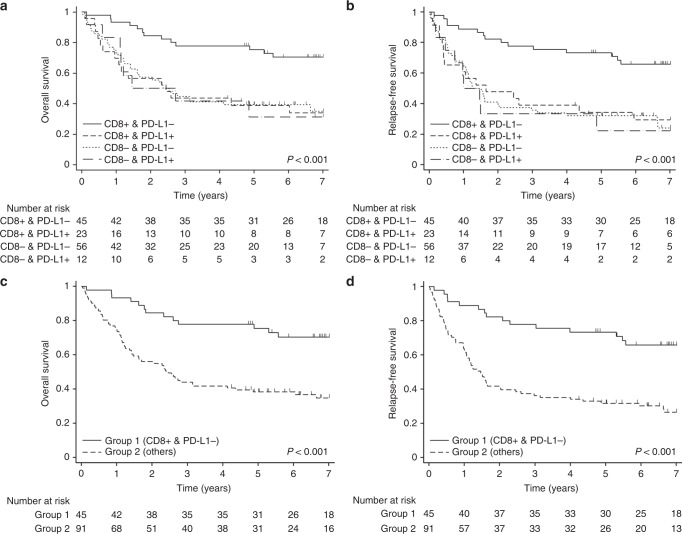
Kaplan–Meier curves for survival based on the expression of CD8/PD-L1 in resectable non-small-cell lung cancer. **a** Overall survival and **b** relapse-free survival in the four stratified groups. **c** Overall survival and **d** relapse-free survival in the dichotomised groups. Group 1 CD8-positive/PD-L1-negative, Group 2 CD8/PD-L1: positive/positive, negative/negative, or negative/positive

**Fig. 4:**
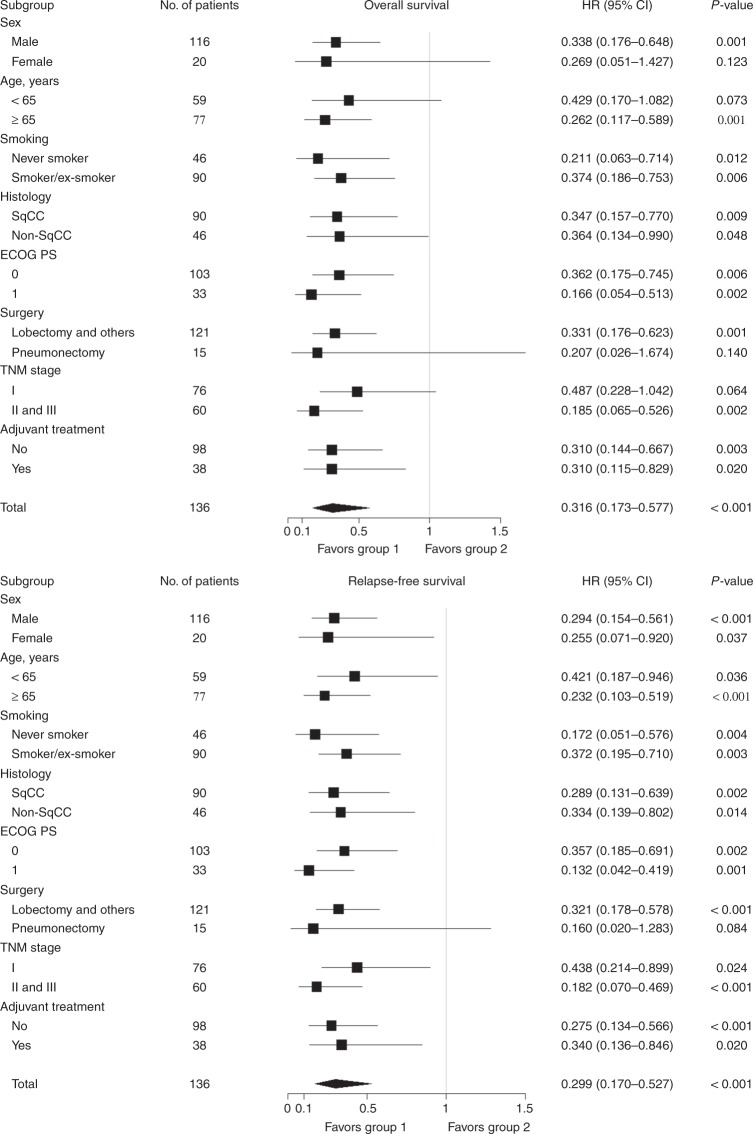
Subgroup analyses for overall and relapse-free survival. HR, hazard ratio; 95% CI, 95% confidence interval; SqCC, squamous cell carcinoma; Non-SqCC, non-squamous cell carcinoma; ECOG PS, Eastern Cooperative Oncology Group performance status; Group 1 CD8-positive/PD-L1-negative, Group 2 CD8/PD-L1: positive/positive, negative/negative, or negative/positive

We also evaluated whether the combined model of CD8 and PD-L1 more accurately predicts the prognosis of patients with resectable NSCLC. Patients with CD8-positive/PD-L1-negative had the highest survival probability, while the other three groups showed similarly low survival probabilities (Fig. 3a, b). Based on these results, we divided the patients into two groups and compared survival rates: group 1 (CD8-positive/PD-L1-negative) versus group 2 (CD8/PD-L1-positive/positive, negative/negative, negative/positive). Group 1 (median OS, NR; 95% CI, NR–NR) showed a significant increase in OS compared with group 2 (median OS, 29.405 months; 95% CI, 17.019–52.238; *p* < 0.001, Fig. 3c). RFS was also significantly increased in group 1 (median RFS, NR; 95% CI 66.793 months—NR) compared with group 2 (median RFS, 17.577 months; 95% CI 12.649–24.345; *p* < 0.001, Fig. 3d). Subgroup analyses for OS and RFS were generally consistent with the analyses of the entire cohort (Fig. 4).

Univariate and multivariate analyses for survival are shown in Table 2. Univariate analysis revealed that age < 65 years, non-SqCC histology, stage I, and group 1 were favourable prognostic factors for OS. Stage I and group 1 were favourable prognostic factors for RFS. The multivariate analysis indicated that age < 65 years, stage I, and group 1 (HR 0.329; 95% CI 0.175–0.619; *p* = 0.001) were independent favourable prognostic factors for OS. In the analysis for RFS, stage I and group 1 (HR, 0.293; 95% CI 0.163–0.527; *p* < 0.001) were independent favourable prognostic factors.

**Table 2:** Cox regression model for overall and relapse-free survival

	Overall survival						Relapse-free survival					
	Univariate			Multivariate			Univariate			Multivariate		
Variables	HR	95% CI	*p*	HR	95% CI	*p*	HR	95% CI	*p*	HR	95% CI	*p*
*Gender*												
Male	Ref.						Ref.					
Female	0.526	0.241–1.149	0.107				0.814	0.431–1.540	0.527			
*Age (years)*												
≥65	Ref.			Ref.			Ref.					
<65	0.591	0.362–0.968	0.036	0.537	0.319–0.902	0.019	0.717	0.456–1.129	0.151			
*Smoking*												
Smoker/ex-smoker	Ref.						Ref.					
Never smoker	0.929	0.565–1.528	0.772				0.934	0.585–1.492	0.776			
*Histology*												
SqCC	Ref.			Ref.			Ref.			Ref.		
non-SqCC^a^	0.520	0.301–0.898	0.019	0.897	0.494–1.628	0.720	0.652	0.398–1.066	0.088	1.010	0.599–1.704	0.970
*ECOG PS*												
1	Ref.						Ref.					
0	0.749	0.444–1.233	0.247				0.811	0.496–1.326	0.404			
*Surgery*												
Pneumonectomy	Ref.						Ref.					
Lobectomy and others^b^	0.842	0.418–1.694	0.629				0.848	0.437–1.646	0.626			
*TNM stage*												
II and III	Ref.			Ref.			Ref.			Ref.		
I	0.613	0.384–0.976	0.039	0.530	0.321–0.874	0.013	0.641	0.413–0.995	0.047	0.619	0.393–0.975	0.039
CD8/PD-L1												
Group 2	Ref.			Ref.			Ref.			Ref.		
Group 1	0.316	0.173–0.577	<0.001	0.329	0.175–0.619	0.001	0.299	0.170–0.527	<0.001	0.293	0.163–0.527	<0.001

*HR* hazard ratio, *95% CI* 95% confidence interval, *SqCC* squamous cell carcinoma, *Non-SqCC* non-squamous cell carcinoma, *ECOG PS* Eastern Cooperative Oncology Group performance status, *Group 1* CD8-positive/PD-L1-negative, *Group 2* CD8/PD-L1: positive/positive, negative/negative, or negative/positive

^a^Including adenocarcinoma, large cell carcinoma, bronchoalveolar carcinoma, and non-small cell carcinoma

^b^Bilobectomy (*n* = 1) and sleeve operation (*n* = 1)

## Discussion

In this study, we investigated the prognostic significance of CD8 and PD-L1 expression in patients with resectable NSCLC using TMAs. The combination of CD8-positive and PD-L1-negative expression (group 1) was significantly associated with favourable OS and RFS. Multivariate analysis demonstrated that group 1 characteristics constituted one of the independent favourable prognostic factors.

Cancer immunoediting, which is the result of immune-surveillance, immune equilibrium, and immune evasion of the immune system, can modify the characteristics of cancer.^26^ Cancer cells are gradually able to gain several immune evasion mechanisms during cancer progression.^27^ Cancers develop various strategies to evade host immune responses, including reduced expression of major histocompatibility complex molecules, loss of tumour antigens, inadequate co-stimulation of T-cells, production of immunosuppressive mediators such as TGF-β, recruitment of immunosuppressive inflammatory cells such as regulatory T cells and myeloid-derived suppressor cells, and expression of immune inhibitory ligands such as PD-L1.^28,29^

PD-L1 is often upregulated on tumour cells and impairs T cell responses, leading to anergy, exhaustion, and apoptosis on engagement with its cognate co-inhibitory receptor PD-1, which is often highly expressed on tumour-infiltrating lymphocytes (TILs).^30–35^ In contrast, CD8+T cells exhibit marked cytotoxic capacities that may induce tumour cell death^36^ by releasing perforins and granzymes in acquired immune responses, thereby playing a critical role in antitumour immunity.^37^ Indeed, CD8+T cells are most likely to be functionally relevant in NSCLC, as the number of apoptotic tumour cells is significantly higher in tumours with a high number of CD3+ and CD8+T cells.^38^ Therefore, CD8+T lymphocytes comprise a well-established group of effector T cells with potent cytotoxic effects in cancer.^39^ In addition, PD-L1-negative tumour cells promote tumour-reactive CD8+T-cell infiltration and proliferation, increased cytokine production, and enhanced cytolytic activity.^40^ Our results showing the positive impact of CD8-positive/PD-L1-negative expression on survival of patients with resectable NSCLC support these theoretical considerations.

Previous studies for PD-L1/CD8 expression in locoregional NSCLC reported various and different results compared with our study. A study that included patients with surgically resected stage I NSCLC showed that CD8+TILs, but not PD-L1, was associated with increased disease-free survival (DFS) and OS.^13^ In another study showing similar findings, a positive impact of CD8+ expression on survival of resected NSCLC patients was shown only in those with low PD-1-to-CD8 ratio.^21^ A French study for patients with surgically treated basaloid squamous cell lung carcinoma (stage I–II, 68%) reported that patients with high PD-L1 expression together with increased PD-1+ and CD8+TILs were associated with an 87% reduction of death risk compared to those with low expression of all three markers.^22^ A Chinese study for patients with *EGFR*-mutated and *ALK*-rearranged NSCLC (stage IA-IIIA, 52%) suggested that OS was shorter in patients with PD-L1+/ CD8+ than in those with PD-L1- / CD8+.^23^ In contrast, another Chinese study showed that neither PD-L1 or CD8 nor their combination were associated with OS in patients with stage I-II NSCLC who underwent surgical resection.^24^ The complex heterogeneity of the tumour microenvironment (TME) may be related to the inconsistency between studies, including the present study. Thommen et al. demonstrated that the immunologic function and metabolism of intratumoural CD8+T lymphocytes differ according to PD-1 expression, and the presence of TILs with high PD-1 expression is correlated with an improved response to PD-1 blockade and with increased OS in NSCLC.^9^ Teng et al. proposed that the TME be stratified into four types based on T-cell infiltration and PD-L1 as follows: type I, PD-L1+TIL+; type II, PD-L1- TIL-; type III, PD-L1+TIL-; and type IV, PD-L1- TIL+.^41^ According to this classification, group 1 in the present study may be relevant to type IV TME. Non-PD-1/PD-L1 suppression pathways, such as myeloid-derived suppressor cells and M2 macrophages, are related to tolerance to PD-1/PD-L1 inhibitors in this type.^41^ If the patients relevant to type IV TME in other previous studies had stronger non-PD-1/PD-L1 suppression pathways than those in the present study, the inconsistency between the present and previous studies can be explained. Collectively, we believe that other components of the TME should be combined with the predictive model to elucidate the prognostic impact of CD8 and PD-L1 expression in future studies.

As with all studies, this work has several limitations that should be taken into consideration. First, the sample size was relatively small for generalising the clinical significance of the expression of CD8 and PD-L1 in resectable NSCLC. Second, there was potential selection bias derived from the retrospective nature of this study. Confirmation in the external validation cohort or large prospective study is required to demonstrate our findings. Third, tissue specimens used in this study were not recently obtained. However, given that the paradigm for perioperative therapy of resectable NSCLC has not been largely changed during last 10 years, our cohort may be enough to assess the clinical outcome of locoregional NSCLC patients. Fourth, the optimal cut-off value for CD8 positivity has not been established. While some studies used the proportion of CD8-positive cells,^13,23,24^ others including this study determined the cut-off value of CD8 by its absolute number.^22,42^ Statistical parameters used to identify the cut-off value were also various, including mean, median, quartile, and previously reported values.^13,22–24,42,43^ Subsequent analyses will be necessary to establish definitive cut-off value for CD8 positivity. Fifth, PD-L1 expression was determined using particular PD-L1 detection antibodies and IHC. However, each company uses a different PD-L1 detection antibody, making it difficult to compare data across clinical trials.^44^ Subsequent analyses will be necessary for standardisation of the PD-L1 antibody.

In conclusion, we suggest the possibility that CD8-positive/PD-L1-negative expression may be an independent favourable prognostic factor for OS and RFS in patients with resectable NSCLC. These findings may be useful to identify patients who are able to be included in a future trial for perioperative immunotherapy in resectable NSCLC. Given several limitations of this study showing inconsistent result compared with previous ones, further large prospective studies regarding CD8, PD-L1, and other biomarkers for the TME should be performed to validate our findings.

## Supplementary information

Immunohistochemical staining for PD-L1 expression

## **Data availability:**

All data are available via the corresponding author.
