# Supplementary material for: Prognostic impact of CD8 and programmed death-ligand 1 expression in patients with resectable non-small cell lung cancer
Source: Br J Cancer. 2019 Feb 12;120(5):547–54. doi: 10.1038/s41416-019-0398-5 (PMC6461857; doi:10.1038/s41416-019-0398-5)
Supplement: Supplementary file 1 — Immunohistochemical staining for PD-L1 expression [file 41416_2019_398_MOESM1_ESM.pptx]

## Slide 1
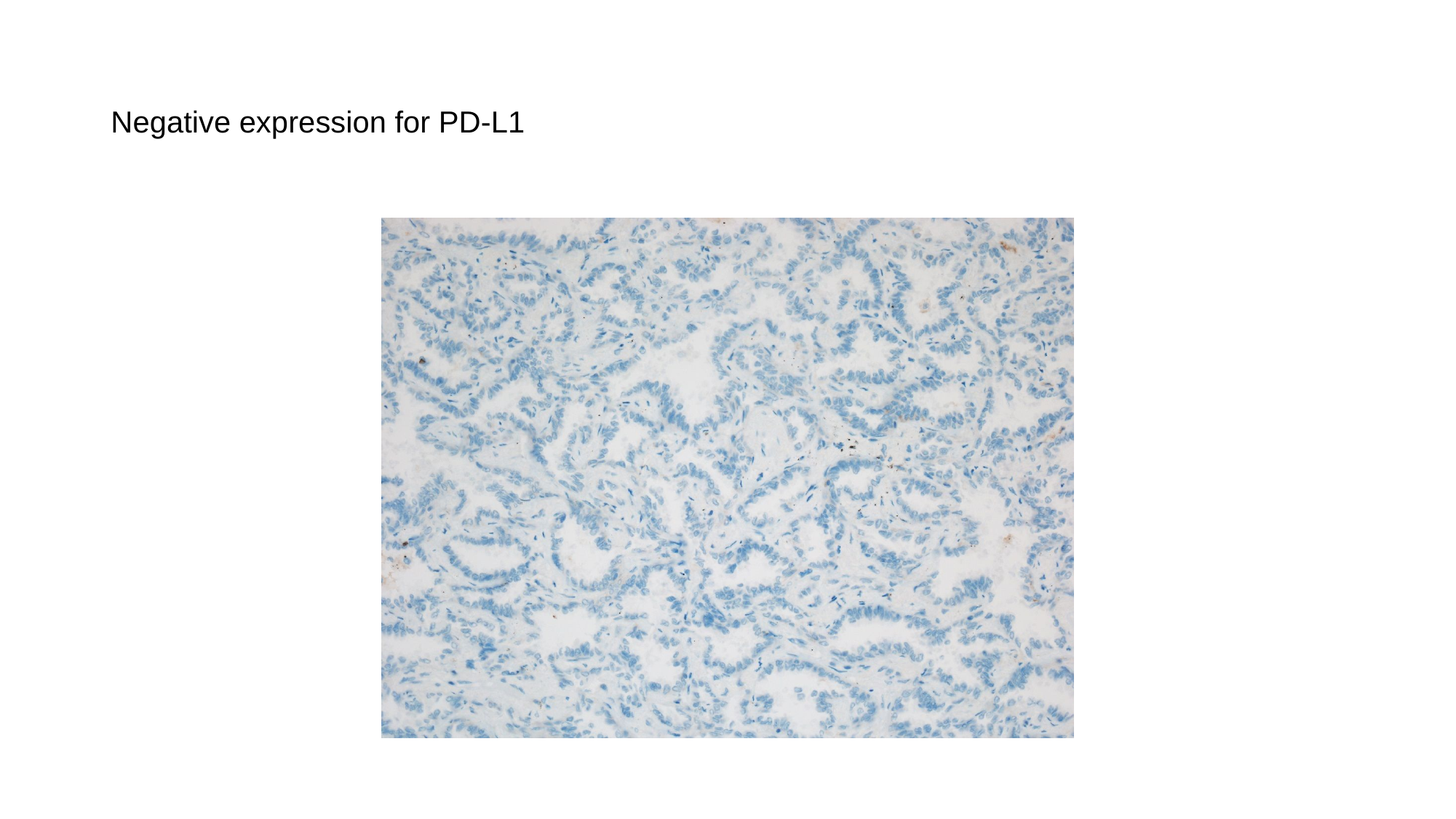

# Negative expression for PD-L1

## Slide 2
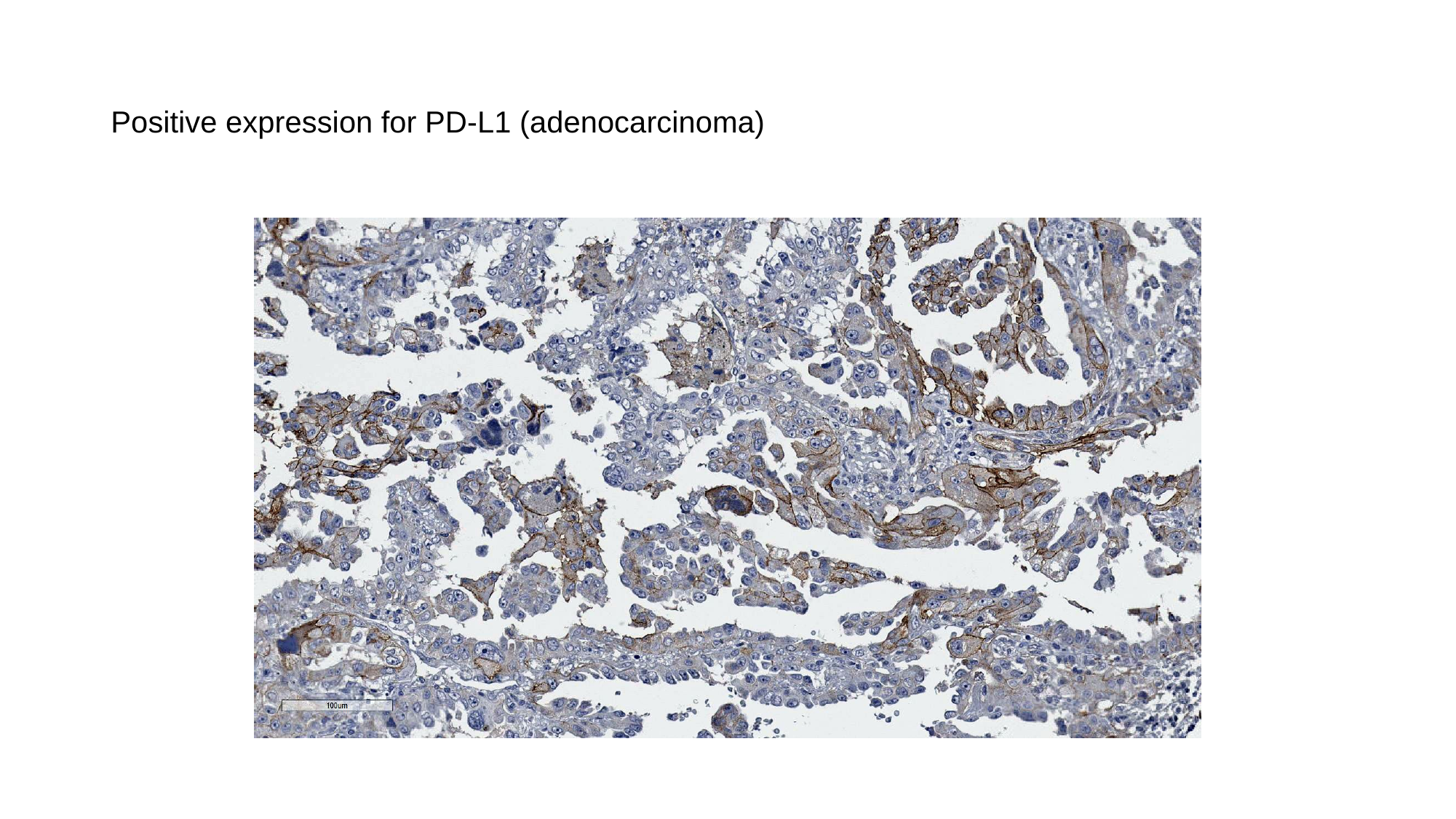

# Positive expression for PD-L1 (adenocarcinoma)

## Slide 3
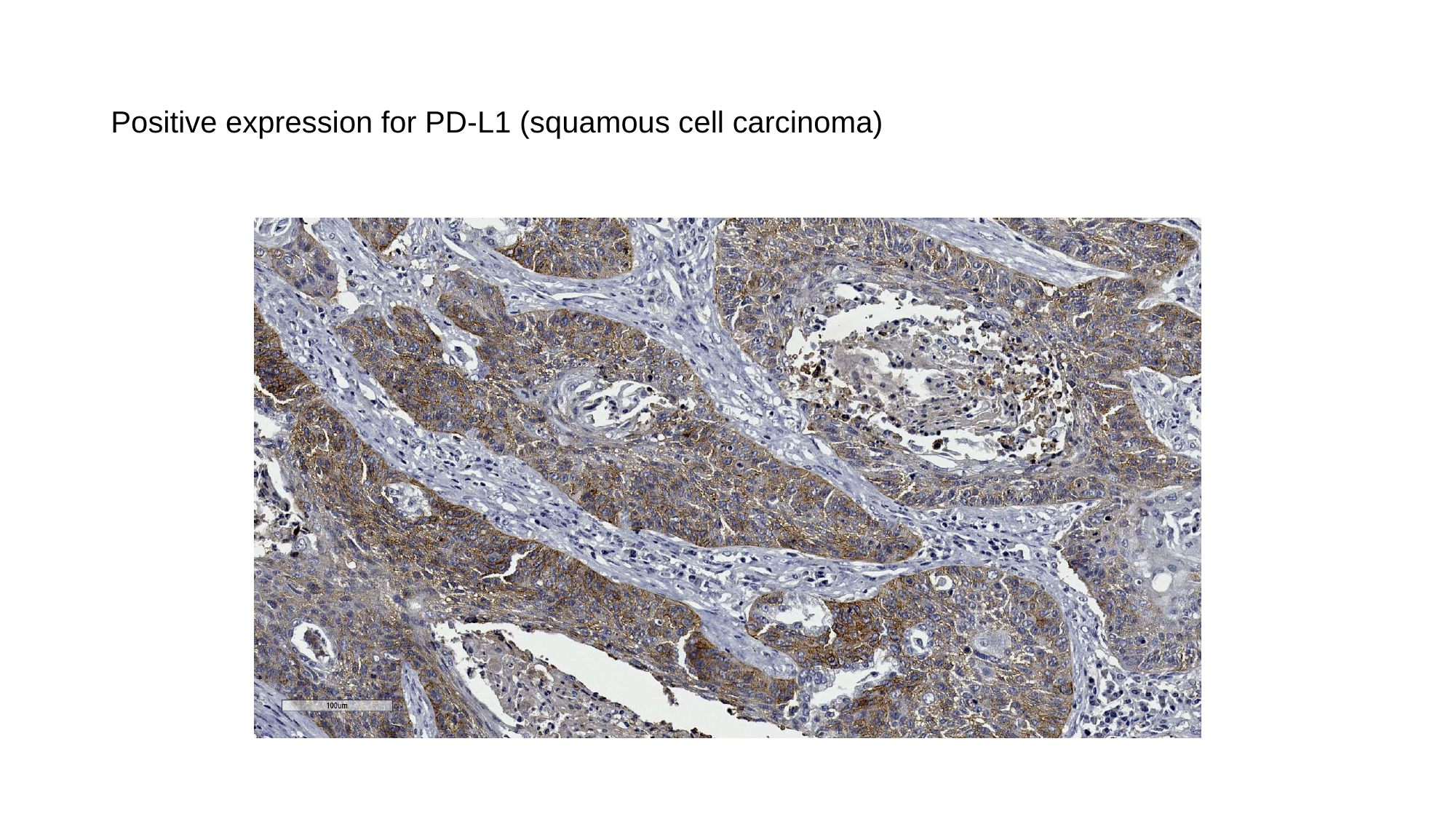

# Positive expression for PD-L1 (squamous cell carcinoma)
